# Identification of the Catechin Uptake Transporter Responsible for Intestinal Absorption of Epigallocatechin Gallate in Mice

**DOI:** 10.1038/s41598-019-47214-4

**Published:** 2019-07-29

**Authors:** Shunsuke Ishii, Hidefumi Kitazawa, Takuya Mori, Aya Kirino, Shun Nakamura, Noriko Osaki, Akira Shimotoyodome, Ikumi Tamai

**Affiliations:** 1 Biological Science laboratories, Kao Corporation, Tochigi, Japan; 2 Analytical Science laboratories, Kao Corporation, Tochigi, Japan; 3 Faculty of Pharmaceutical Sciences, Institute of Medical, Pharmaceutical and Health Sciences, Kanazawa University, Kanazawa, Japan

**Keywords:** Drug delivery, Transporters

## Abstract

Many studies have shown that epigallocatechin gallate (EGCg) contribute to the health benefits of green tea, although its bioavailability is usually low. However, the mechanism underlying its intestinal absorption remains unclear. In human subjects, it has been reported that the bioavailability of EGCg increases after repeated oral catechin intake. We hypothesized that a certain uptake transporter was involved in this increase, and investigated a novel EGCg transporter. We first confirmed the increase in EGCg bioavailability in mice fed the catechin diet for two weeks. Then, *in situ* intestinal catechin infusion exhibited that the absorption of EGCg in the ileum was selectively increased in mice fed the catechin diet. A comprehensive analysis of plasma membrane proteins revealed 10 candidates for EGCg transporter, which were selectively increased in the ileum. EGCg uptake by a *Xenopus laevis* oocyte expressed with respective transporter revealed that oocytes microinjected with DTDST cRNA exhibited significantly higher EGCg uptake. Furthermore, uptake of EGCg by CHO-K1 cells stably expressing DTDST was significantly higher than that by mock cells, which was nullified by treating with a DTDST inhibitor. In conclusion, this study identified DTDST as a novel intestinal EGCg transporter that is upregulated after repeated oral catechin intake.

## Introduction

Green tea is a popular beverage worldwide and has many beneficial health effects, including anti-cancer, cardioprotective, anti-inflammatory, anti-diabetic, and anti-obesity effects^1–4^. Catechins are a family of polyphenols found at high concentrations in green tea, and many studies have shown that several catechins, particularly epigallocatechin gallate (EGCg), contribute to the health benefits of green tea^5–11^. It is thought that the physiological activity of EGCg depends on its bioavailability, which is usually poor^12–14^; therefore, increasing the bioavailability of EGCg may improve its health benefits and consequently those of green tea.

Since catechins are small, water-soluble molecules, it has been suggested that catechins are absorbed via the paracellular pathway in the small intestine^15^. However, it is also known that efflux transporters such as multidrug resistance protein 1 (MDR1) and multidrug resistance-associated protein 2 (MRP2) transport many kinds of polyphenols from inside of enterocytes to the intestinal lumen^16,17^, and some studies have demonstrated that EGCg and other catechins are excreted via MRP2^18–21^. Thus, it is likely that both the paracellular and transcellular pathways are involved in the absorption of EGCg.

It was recently reported that several transporters are capable of transporting polyphenols into cells rather than pumping them out. For example, quercetin has been shown to be absorbed by organic anion transporting polypeptides (OATPs) and organic cation transporter 1^22,23^. OATP1A2 and OATP1B3 are also potential EGCg transporters^24^, while they are not expressed in the intestine. Thus, the mechanism responsible for intestinal absorption of EGCg and other catechins is yet to be fully clarified.

In human subjects, plasma levels of EGCg, but not those of other catechins such as epigallocatechin (EGC) and epicatechin (EC), are significantly increased after 4 weeks of repeated oral catechin intake^25^. Since this observation suggests that there might be a unique metabolic pathway or a specific absorption mechanism for EGCg, we hypothesized that a certain uptake transporter in the gastrointestinal tract is responsible for the reported increase in EGCg bioavailability. Here, we identified an EGCg transporter by examining changes in EGCg bioavailability after repeated catechin intake in mice.

## Materials and Methods

### Materials

Polyphenon 70 S containing 39.9% EGCg, 24.7% EGC, 10.9% epicatechin gallate (ECg), 9.8% EC, 7.5% gallocatechin (GC), 4.0% gallocatechin gallate (GCg), 2.2% catechin (C), and 1.0% catechin gallate (Cg) was purchased from Mitsui Norin (Tokyo). Purified EGCg was from Nagara Science (Gifu, Japan). Sulfatase (from abalone entrails, Type VIII) and β-glucuronidase (from *Escherichia coli*, Type IX-A) were from Sigma Aldrich Japan (Tokyo). All other chemicals were commercially available materials at the highest grade.

### Animals

Seven-week-old male C57BL/6 J mice were purchased from CLEA Japan (Tokyo) and group housed (four mice per cage). Room temperature was maintained at 23 ± 2 °C with a humidity of 45–65%. Mice were exposed to a 12-h light/dark cycle and water was freely accessible throughout the study. All animal experiments were conducted in the experimental animal facility of the Kao Tochigi Institute. Animal studies have been carried out in accordance with the Guide for the Care and Use of Laboratory Animals as adopted and promulgated by the U.S. National Institutes of Health. The Animal Care Committee of Kao Corporation approved the protocols used in the present study (protocol number N2014-0016A).

Mice were fed normal chow (CE-2; CLEA Japan) for one week and then randomly divided into two groups: a normal diet group (control group) and a catechin diet group (catechin group) (Table 1). Mice were fed each test diet *ad libitum* for two weeks and then subjected to oral administration testing (n = 5/group), *in situ* catechin intestinal infusion testing (n = 3 to 6/group), or proteomics analysis (n = 3/group).

**Table 1:** Test diet composition (%).

	Normal diet (Control group)	Catechin diet (Catechin group)
Corn oil	10	10
Casein	20	20
Cellulose	4	4
Mineral mixture	3.5	3.5
Vitamin mixture	1	1
Potato starch	61.5	61.0
Polyphenon 70 S	0	0.5
Total	100	100

### Oral administration study in mice

After two weeks of feeding with the normal or catechin diet, mice were fasted for 16 h and orally administered catechin solution (100 mg/kg body weight [bw] of polyphenon 70 S). Under anesthesia with isoflurane (Forane; AbbVie GK, Tokyo), blood samples were collected from the abdominal aorta at 0, 20, 50, 90, 180, 300, and 720 min after catechin administration and immediately centrifuged (11,000 × *g* for 10 min at 4 °C) to obtain plasma samples. One-tenth volume of stabilization buffer (0.4 M NaH_2_PO_4_, 20% ascorbic acid, 0.1% ethylenediaminetetraacetic acid; pH 3.6) was added to the plasma samples to suppress catechin degradation, and the samples were then kept at −80 °C until use.

### *In situ* intestinal infusion study in mice

On the final day of the two-week feeding period with the normal or catechin diet, mice were fasted for 16 h and then anesthetized with isoflurane. Under anesthesia, the mice were maintained at body temperature with a heating pad. The small intestine was exposed by abdominal incision and catechin solution (100 mg/kg-bw of polyphenon 70 S) was infused via a syringe with a 27 G needle (Terumo, Tokyo) into four different parts of the gastrointestinal tract: pylorus, jejunum (5 cm inferior to the pylorus), ileum (10 cm superior to the cecum), or cecum (1 cm superior to the cecum). Before infusing the catechin solution, a ligature was applied close to the infusion point to prevent backflow of the catechin solution. Blood samples were collected from the orbital venous plexus at 0, 15, and 30 min after infusion. Plasma samples were obtained and stored in the same manner as in the oral administration study.

### Preparation of plasma membrane proteins in intestinal epithelial cells of mice

After the two-week feeding period with the normal or catechin diet, mice were fasted for 16 h and the jejunum (0–3.5 cm inferior to the pylorus) and ileum (5–10 cm superior to the cecum) were resected under anesthesia with isoflurane. Intestinal epithelial cells were obtained by scraping the cut-open intestinal tracts on ice. Plasma membrane protein was purified without detergent using a Minute Plasma Membrane Protein Isolation Kit (Invent Biotechnologies, Plymouth, MN) in accordance with the manufacturer’s instructions. Purified membrane protein was dissolved in 200 μL of phase transfer surfactant buffer (12 mM sodium deoxycholate, 12 mM sodium N-dodecanoylsarcosinate, 100 mM Tris–HCl; pH 9.0) and heated at 95 °C for 5 min followed by sonication for 20 min. Protein concentration was quantified by a Pierce BCA Protein Assay Kit (Thermo Fisher Scientific, Tokyo). The test solution was mixed with one-hundredth volume of 50 mM NH_4_HCO_3_ buffer including 1 M dithiothreitol and incubated for 30 min at room temperature. Subsequently, one-twentieth volume of 50 mM NH_4_HCO_3_ buffer containing 1 M iodoacetamide was added and the samples were incubated for 30 min at room temperature in the dark. Then, the samples were diluted five-fold with 50 mM NH_4_HCO_3_ buffer and digested with Lys-C (1 μg/100 μg-protein) for 3 h at 37 °C and then with trypsin (1 μg/100 μg-protein) overnight at 37 °C. Equal volumes of ethyl acetate and trifluoroacetic acid (0.5% of final concentration) were added to the samples and the solution was mixed well. After centrifugation at 15,700 × *g* for 2 min at 20 °C, the supernatant was removed and dried under reduced pressure at 50 °C. The obtained peptide digest was passed through a GL-Tip SDB column (GL Sciences, Tokyo) and then diluted with water containing 0.1% formic acid and 2% acetonitrile (1 μg/μL).

### Shotgun proteomics analysis using liquid chromatography tandem-mass spectrometry

Preprocessed samples were injected into a high-performance liquid chromatography (HPLC) system (Ultimate 3000 RSLC nano System; Thermo Fisher Scientific), concentrated using an Acclaim PepMap 100 C18 Nano-Trap Column (3 µm beads, 75 µm i.d., 20 mm long; Thermo Fisher Scientific), and then separated by a Mono Cap High Resolution 2000 column (100 µm i.d., 2 m long; GL Sciences). The elution buffer was composed of buffer A (0.1% formic acid) and buffer B (80% acetonitrile containing 0.1% formic acid), and the gradient profile of the first step was a 480-min linear gradient from 5% to 40% buffer B followed by a 10-min linear gradient to 100% buffer B, with 10 min at 100% buffer B, at a flow rate of 500 nL/min. Auto-calibration was performed between each analysis. The outlet was connected to a Triple TOF 5600 + tandem-mass spectrometry system (AB SCIEX, Tokyo) connected to a PicoTip NanoSpray Emitter FS360-50-15-N (New Objective, Woburn, MA). A survey scan for positive ions from m/z 400 to 1000 was initially performed, followed by a tandem-mass spectrometry scan from m/z 100 to 1500 after collision-induced dissociation with data-dependent acquisition. Proteins were identified by cross-referencing the obtained spectrum and the Swiss-Prot protein sequence database, and identified proteins were then categorized using the Ingenuity Pathway Analysis software (Ingenuity Systems, Redwood City, CA). For proteins categorized as plasma membrane transporters, an additional liquid chromatography tandem-mass spectrometry analysis was performed for the same m/z range with data-independent acquisition, and the expression level of each molecule was quantified by its peak area (Peak View; Thermo Fisher Scientific).

### Uptake study by *Xenopus laevis* oocytes

Total RNA was extracted from ileal epithelial cells by an RNeasy mini kit (QIAGEN, Tokyo) and cDNA was synthesized by SuperScript III Reverse Transcriptase (Life Technologies Japan, Tokyo). Open reading frames of candidate genes (Table 2) were amplified by polymerase chain reaction and cloned into pSP64 poly (A) vector (Promega, Tokyo) or modified vectors with another restriction site inserted (Supplemental Table 1). A Kozak sequence at the 5′-terminal and FLAG tag at the 3′-terminal were added to each open reading frame. Constructs were cut into single strands at the designated restriction site and purified by electrophoresis and gel extraction by a MagExtractor PCR & Gel Clean-up kit (Toyobo, Osaka). cRNAs were obtained by *in vitro* transcription by an mMESSAGE mMACHINE SP6 transcription kit (Thermo Fisher Scientific) and purified by a QIAprep Spin Miniprep Kit (QIAGEN). Defolliculated *Xenopus laevis* oocytes were microinjected with 25 ng of each cRNA and incubated at 25 °C in modified Barth solution (88 mM NaCl, 1 mM KCl, 0.33 mM Ca(NO_3_)_2_, 0.41 mM CaCl_2_, 0.82 mM MgSO_4_, 2.4 mM NaHCO_3_, 10 mM HEPES; pH 7.4) containing 50 mg/L gentamycin. After 3 days, oocytes were transferred to modified Barth solution containing 100 or 500 μM EGCg and 1 mM ascorbic acid and then incubated at 23 °C. After 0, 0.5, 1, and 2 h, oocytes were removed and washed with ice-cold modified Barth solution. Samples were kept at −80 °C until catechin quantification.

**Table 2:** EGCg transporter candidates identified by means of a shotgun proteomics analysis.

Protein name	Gene symbol	Entrez gene name	NCBI ref seq
ZIP14	*Slc39a14*	solute carrier family 39 (zinc transporter), member 14	NM_001135151
ASBT	*Slc10a2*	solute carrier family 10 (sodium/bile acid cotransporter), member 2	NM_011388
CTL4	*Slc44a4*	solute carrier family 44, member 4	NM_023557
DRA	*Slc26a3*	solute carrier family 26 (anion exchanger), member 3	NM_021353
DTDST	*Slc26a2*	solute carrier family 26 (anion exchanger), member 2	NM_007885.2
LAT2	*Slc7a8*	solute carrier family 7 (amino acid transporter light chain, L system), member 8	NM_016972
NBC1	*Slc4a4*	solute carrier family 4 (sodium bicarbonate cotransporter), member 4	NM_018760
MNK	*Atp7a*	ATPase, Cu ++ transporting, alpha polypeptide	NM_001109757
KCC3	*Slc12a6*	solute carrier family 12 (potassium/chloride transporter), member 6	NM_133648
MCT1	*Slc16a1*	solute carrier family 16 (monocarboxylate transporter), member 1	NM_009196

### Uptake study by CHO-K1 cells expressing diastrophic dysplasia sulfate transporter

The open reading frame of diastrophic dysplasia sulfate transporter (DTDST) with C-terminal FLAG tag was resected from the pSP64 poly(A) vector and recombined into the pcDNA3.1(+) vector (Thermo Fisher Scientific) by the *Hin*dIII and *Xba*I sites. The construct was purified by an EndoFree Plasmid Maxi Kit (QIAGEN) and transfected using Xfect Transfection Reagent (Clontech Laboratories, Mountain View, CA) into CHO-K1 cells cultured in F-12 medium containing 10% fetal bovine serum. After 48 h, the transfected cells were seeded into 96-well plates in a limiting dilution and cultured in F-12 medium containing 10% fetal bovine serum and 1 mg/mL G418. After selection with neomycin, the cell line with high DTDST gene expression level was used in the following EGCg uptake assay. The cells were preincubated in F-12 medium containing 10% fetal bovine serum overnight. After equilibrating for 1 h in Hanks’ balanced salt solution (pH 6.0), 100 μM EGCg was added to the medium and the cells were incubated at 37 °C for up to 4 h. A DTDST inhibitor, 4,4′-diisothiocyano-2,2′-stilbenedisulfonic acid (DIDS, 100 μM), or vehicle (dimethyl sulfoxide) was added to the cells 1 h before EGCg exposure. In the kinetic analysis, cells were incubated with EGCg or gallic acid at concentrations ranging from 1 to 1000 μM at 37 °C for 30 min. After incubation, the cells were washed twice with ice-cold phosphate-buffered saline and lysed in CelLytic lysis buffer (Sigma-Aldrich Japan). Samples were kept at −80 °C until catechin quantification.

### Catechin quantification

In the oral administration test, catechin in plasma was extracted using ethyl acetate and quantified by liquid chromatography–electrochemical detection. β-Glucuronidase (125 U), sulfatase (0.5 U), and 10 μL of 0.4 M Na_2_PO_4_ buffer (pH 7.4) were added to 100 μL of plasma sample and the mixture was incubated for 45 min at 37 °C. Then, 1 mL of ethyl acetate was added to the sample and the mixture was mixed rigorously for 4 min. After centrifugation (2,400 × *g* for 10 min at 4 °C), the supernatant was removed. Extraction with ethyl acetate was repeated and the supernatants were merged. The supernatants were dried by nitrogen purging and the residues were dissolved in 15% acetonitrile in water. The obtained samples were injected into an HPLC system (2695 Separations Module; Waters, Tokyo) equipped with a Coulochem III electrochemical detector (ESA, Chelmsford, MA) and an Inertsil ODS-2 column (4.6 × 250 nm; GL Sciences). Separation was performed with buffer A (100 mM NaH_2_PO_4_ containing 1.75% acetonitrile and 0.12% tetrahydrofuran, pH 3.35) and buffer B (15 mM NaH_2_PO_4_ buffer containing 58.5% acetonitrile and 12.5% tetrahydrofuran, pH 3.45). The gradient was changed linearly from 96% buffer A and 4% buffer B to 83% buffer A and 17% buffer B (7–25 min), 72% A and 28% B (25–31 min), 67% A and 33% B (31–37 min), 2% A and 98% B (37–38 min), maintained at 2% A and 98% B (38–43 min), and changed to 96% A and 4% B (43–44 min) at a flow rate of 1 mL/min.

In the intestinal infusion test, catechin in plasma was purified by solid phase extraction and measured by HPLC–tandem-mass spectrometry. β-Glucuronidase (62.5 U), sulfatase (0.25 U), 16 μL of 0.4 M phosphate-buffered saline (pH 3.6), and 16 μL of 0.4 M phosphate-buffered saline (pH 7.4) were added to 50 μL of serum samples and incubated for 45 min at 37 °C. Then, 600 μL of 0.2 M acetic acid and 10 ng of ethylgallate (internal standard) was added to the samples and they were loaded onto a 1-cc Oasis HLB cartridge (Waters) preconditioned with water and dimethyl sulfoxide. After washing with water and 30% methanol, catechin was eluted with dimethyl sulfoxide containing 0.1% ascorbic acid. Samples were freeze-dried and dissolved in 50 μL of 10% acetonitrile containing 0.5% ascorbic acid. Samples were injected into an HPLC system (Infinity 1260; Agilent Technologies Japan, Tokyo) equipped with a 3200 QTRAP liquid chromatography tandem mass spectrometry system (AB SCIEX, MA, USA) and an L-column2 ODS column (Chemicals Evaluation and Research Institute, Tokyo). Separation was performed using buffer A (0.1% formic acid solution) and buffer B (acetonitrile). The gradient was linearly changed from 97% buffer A and 3% buffer B to 85% buffer A and 15% buffer B (0–2 min), 81% A and 19% B (2–6 min), 50% A and 50% B (6–6.1 min), maintained at 50% A and 50% B (6.1–9 min), and changed to 97% A and 3% B (9–12 min) at a flow rate of 0.7 mL/min.

In the transport assay using *Xenopus* *laevis* oocytes and CHO-K1 cells, catechins taken up by cells was extracted by ethyl acetate and measured by liquid chromatography–tandem-mass spectrometry. Two oocytes were placed in modified Barth solution and lysed by sonication (Branson Sonifier 150, intensity 4; Emerson Japan, Kanagawa) on ice and then centrifuged (21,000 × *g*) for 10 min at 4 °C. The supernatant was removed and extraction with ethyl acetate was performed in the same manner as described in the paragraph above. Merged samples were dried under low pressure and dissolved in 10% acetonitrile containing 0.5% ascorbic acid. HPLC–mass spectrometry analysis was performed by the same conditions used in the intestinal infusion test.

### Statistical analysis

Data are expressed as mean ± SD. Time-course data were compared by two-factor repeated-measures ANOVA to evaluate the group-by-time interaction. In cases where a significant diet-by-time interaction was observed, an intergroup comparison at each time point was subsequently performed by an unpaired Student’s *t*-test. All other statistical comparisons were made using a Student’s *t*-test. A *P*-value of less than 0.05 was considered significant. Statistical analyses were performed using SPSS version 18 (IBM, Armonk, NY). The kinetic parameters of DTDST-mediated EGCg uptake were obtained by fitting of the data to Michaelis–Menten equation in the enzyme kinetics module of GraphPad Prism software Version 6.0 (GraphPad software, La Jolla, CA).

## Results

### Effect of repeated intake of catechins on their *in vivo* absorption in mice

**Figure 1:**
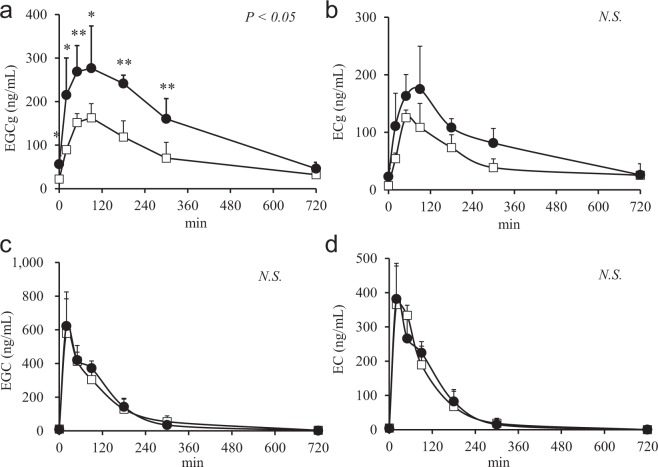
Plasma concentration of (**a**) epigallocatechin gallate (EGCg), (**b**) epicatechin gallate (ECg), (**c**) epigallocatechin (EGC), and (**d**) epicatechin (EC) after oral catechin administration (catechin group, ●; control group, □; n = 5/group). Two-factor repeated measures ANOVA was used to evaluate the group-by-time interaction and the *P* value for this test is shown in the upper-right of each panel. Data are presented as mean ± SD. Significant differences as determined by Student’s *t*-test are indicated by *(*P* < 0.05) and **(*P* < 0.01).

We first examined whether the bioavailability of EGCg was increased by repeated catechin intake in mice. Plasma levels of four catechin species (EGCg, ECg, EGC, EC) were elevated immediately after oral administration of catechin solution, reaching a peak concentration at 20 min for EGC and EC, and at 90 min for EGCg and ECg (Fig. 1). Mice fed the catechin diet for two weeks exhibited a significantly higher plasma EGCg level from 0 to 300 min after oral administration of catechin solution compared with mice fed the control diet (Fig. 1a); however, there were no significant differences in the plasma concentration of other catechin species between the two groups (Fig. 1b–d).

### Intestinal regional specificity of absorption by *in situ* intestinal catechin infusion in mice

**Figure 2:**
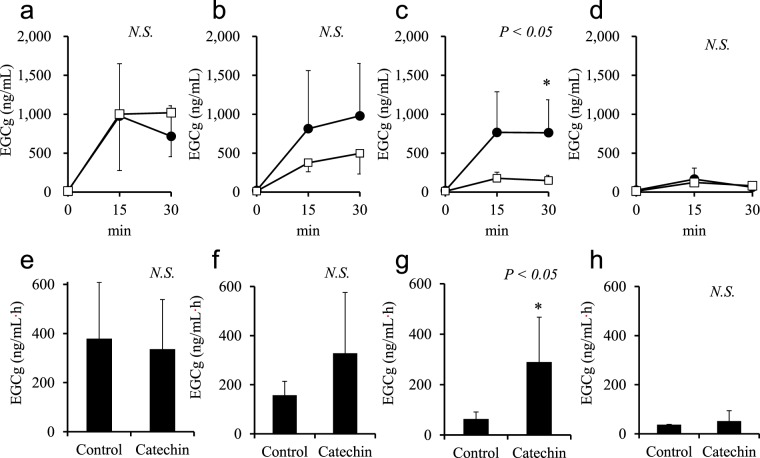
Plasma concentrations of epigallocatechin gallate (EGCg) after *in situ* catechin infusion into (**a**) the pylorus, (**b**) jejunum, (**c**) ileum, or (**d**) cecum (catechin group, ●; control group, □; n = 3–6/group). Two-factor repeated measures ANOVA was used to evaluate the group-by-time interaction and the *P* value for this test is shown in the upper-right of each panel. The area under the curve is shown for (**e**) the pylorus, (**f**) jejunum, (**g**) ileum, and (**h**) cecum. Data are presented as mean ± SD. Significant differences as determined by Student’s *t*-test are indicated by *(*P* < 0.05).

Next, an *in situ* intestinal catechin infusion study was performed to determine the site in the small intestine where the increase in plasma EGCg occurred in the mice fed the catechin diet. The plasma EGCg level in the control group was highest in the mice administered catechin in the pylorus and gradually decreased as the site of catechin administration was moved toward the distal part of the intestine (Fig. 2). In mice administered catechin in the ileum, the plasma EGCg level was significantly higher in the catechin group than in the control group at 30 min after administration (Fig. 2c), whereas it remained comparable between the two groups in mice administered catechin in the pylorus, jejunum, or cecum (Fig. 2a,b,d). A significant increase in the area under the curve of plasma EGCg was observed only in mice in which catechin solution was injected into the ileum (Fig. 2e–h).

### Screening of responsible transporter molecules for increased EGCg absorption after an intake of catechins

To elucidate the transporter molecules involved in the plasma EGCg increase in mice fed the catechin diet, a comprehensive shotgun proteomics analysis of plasma membrane proteins expressed in the jejunum and ileum of mice fed the control diet or the catechin diet was performed. A total of 371 membrane proteins including 82 transporters were identified in the analysis (Supplemental Table 2). The expression levels of each protein were compared between the two groups and the 10 molecules shown in Table 3 were selected as EGCg transporter candidates based on two inclusion criteria: 1) ileal expression of the protein in the catechin group was more than 2-fold of the expression in the control group, and 2) the difference in jejunal expression of the protein was less than 1.5-fold between the catechin group and the control group.

**Table 3:** Fold change of intestinal expression levels of EGCg transporter candidates.

Protein name	UniProtKB ID	Fold Change*	
		Jejunum	Ileum
ZIP14	S39AE_MOUSE	1.1	13.4
ASBT	NTCP2_MOUSE	0.8	7.1
CTL4	CTL4_MOUSE	0.5	4.9
DRA	S26A3_MOUSE	1.4	3.0
DTDST	S26A2_MOUSE	1.3	3.0
LAT2	LAT2_MOUSE	1.2	2.8
NBC1	S4A4_MOUSE	1.5	2.8
MNK	ATP7A_MOUSE	1.5	2.1
KCC3	S12A6_MOUSE	1.4	2.0
MCT1	MOT1_MOUSE	1.1	2.0

*Fold Change of the average expression levels in the catechin group to the control group (n = 3/group).

### Uptake study by *Xenopus* oocytes expressing selected transporters

**Figure 3:**
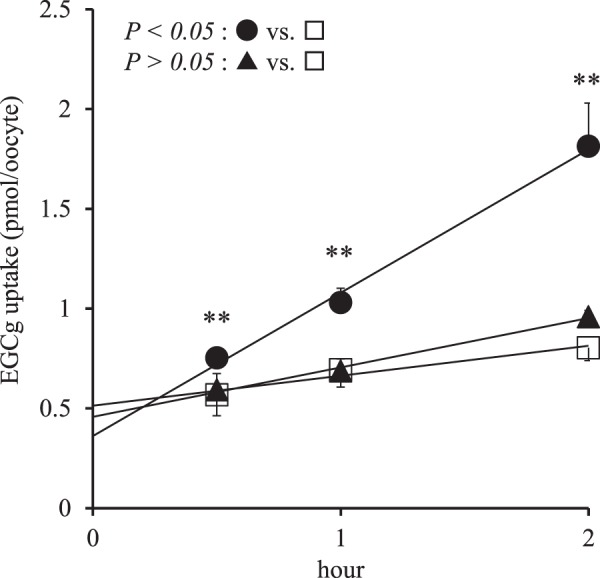
Time-course data of epigallocatechin gallate (EGCg) uptake by *Xenopus laevis* oocytes microinjected with diastrophic dysplasia sulfate transporter (DTDST) cRNA (●), zinc transporter 14 (ZIP14) cRNA (▲) or water (□) (n = 6–8/group) incubated with 500 μM EGCg. Linear approximation was performed and the line is shown. Two-factor repeated measures ANOVA was used to evaluate the group-by-time interaction and the *P* values are shown in the upper-left of the panel. Data are presented as mean ± SD. Significant differences as determined by Student’s *t*-test are indicated by **(*P* < 0.01).

**Figure 4:**
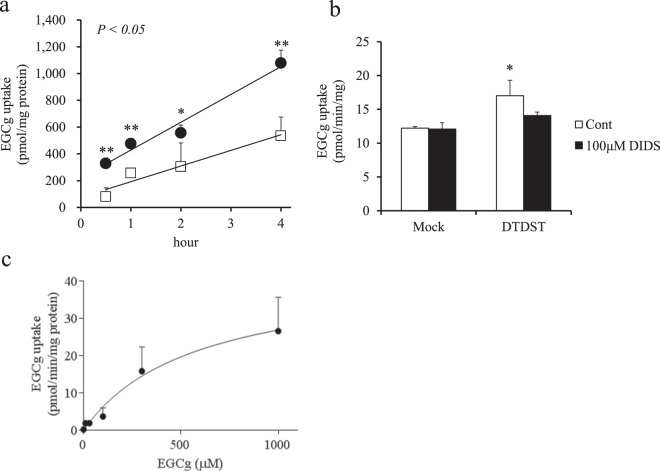
(**a**) Time-course data of epigallocatechin gallate (EGCg) uptake by CHO-K1 cells stably expressing diastrophic dysplasia sulfate transporter (DTDST; ●) or mock cells (□) (n = 4/group) incubated with 500 μM EGCg. Linear approximation was performed and the line is shown. Two-factor repeated measures ANOVA was used to evaluate the group-by-time interaction and the *P* value is shown in the upper-left of the panel. Data are presented as mean ± SD. Significant differences as determined by means of Student’s *t*-test are indicated by *(*P* < 0.05) and **(*P* < 0.01). (**b**) EGCg uptake by CHO-K1 cells stably expressing DTDST or mock cells after treatment with the DTDST inhibitor 4,4′-diisothiocyano-2,2′-stilbenedisulfonic acid (DIDS) or vehicle (n = 3/group) incubated with 500 μM EGCg for 30 min. Data are presented as mean ± SD. Significant differences as determined by Student’s *t*-test are indicated by *(*P* < 0.05). (**c**) Kinetics of EGCg uptake mediated by DTDST after subtracting the values obtained with mock cells (n = 4). CHO-K1 cells stably expressing DTDST or mock cells were incubated with EGCg at concentrations ranging from 1 to 1000 μM for 30 min. Data are presented as mean ± SD. The kinetic parameters were obtained by fitting the data to the Michaelis–Menten equation in the enzyme kinetics module of GraphPad Prism software Version 6.0.

EGCg uptake by 10 selected proteins was examined using an oocyte expression system, and oocytes microinjected with either DTDST cRNA or ZIP14 cRNA exhibited significantly higher EGCg uptake (Supplemental Fig. 1). Next, a time-dependent uptake of EGCg was confirmed in oocytes microinjected with DTDST cRNA (Fig. 3). However, ZIP14 was not studied further, since the group-by-time interaction was not significant between oocytes microinjected with ZIP14 cRNA and mock cells (Fig. 3). The uptake rate of EGCg by DTDST-expressing oocytes obtained from the slope of the time course (0.718 pmol/hour/oocyte, Fig. 3) was 4.8-times higher than that for the control oocytes (0.150 pmol/hour/oocyte). The gene encoding DTDST was subsequently transfected into CHO-K1 cells and a stable cell line was cloned for the following analysis of the DTDST-mediated transport of EGCg. Although time-dependent EGCg uptake was observed both in mock and DTDST-expressing cell lines, it was significantly higher in DTDST-expressing cells at all time points examined (Fig. 4a). The uptake rate obtained from the slope of the time course for the DTDST-expressing clone (208 pmol/hour/mg protein) was 1.8-fold of that for mock cells (117 pmol/hour/mg protein). In addition, the significantly higher uptake by the DTDST-expressing clone compared with mock cells was nullified in the presence of the DTDST inhibitor DIDS (Fig. 4b). A kinetic analysis based on dose response provided an estimated K_m_ of 576 ± 244 μM and V_max_ of 42.3 ± 8.4 pmol/min/mg-protein for DTDST-mediated EGCg transport (Fig. 4c). Although the transport activity for gallic acid was also evaluated, no differences were observed between CHO-K1 cells stably expressing DTDST and mock cells (Supplemental Fig. 2).

## Discussion

In the present study, we investigated the mechanism for the intestinal absorption of catechins based on the reported pharmacokinetic characteristics that exhibited increased bioavailability of EGCg after repeated intake of catechins in humans^25^.

First, we studied *in vivo* effect of repeated intake of catechin diet on the absorption of several catechins in mice. As clearly shown in Fig. 1, plasma concentration of only EGCg was increased after oral administration in repeated catechin diet-treated mice. Accordingly, the pharmacokinetic changes observed in humans upon catechin ingestion were reproduced in mice, so this model was used for the following study. Regarding the mechanism for this increased bioavailability of EGCg, we hypothesized that a certain intestinal uptake transporter could be upregulated by a catechin-containing diet. Based on this hypothesis, such upregulated proteins were searched by a comprehensive shotgun proteomics analysis of intestinal tissues. To roughly screen such proteins, intestinal absorption of EGCg was first evaluated after dividing mouse whole intestines to four sections independently with and without a 2-week catechin diet. To evaluate EGCg absorption in different parts of the intestine, we initially attempted a traditional *ex vivo* analysis approach, but it was difficult to detect changes in EGCg absorption. To address this issue, we developed a technique called *in situ* intestinal catechin infusion that allowed us to evaluate EGCg absorption in specific parts of the intestine. By our novel approach, it was found that EGCg was absorbed in the upper intestine (Fig. 2), consistent with the results of the oral administration test that showed an early elevation of plasma EGCg level after oral administration of catechin. Increased absorption of EGCg by the catechin-containing diet was observed only in the ileum, therefore this part of intestine was further used for a comprehensive shotgun proteomics analysis. Among transporter-like proteins that showed selectively increased expression in the ileum, 10 proteins were obtained (Table 3) and only DTDST exhibited a significant time-dependent increase of EGCg uptake in heterologous expression in *Xenopus* oocytes and CHO-K1 cells (Figs 3 and 4).

DTDST has been known as a sulfate transporter encoded by the *Slc26a2* gene, which transports inorganic ions such as sulfate, chloride, and hydroxide^26,27^. Recently, it was also reported that an organic substrate, oxalate, was transported by DTDST, implicating the broader selectivity of this transporter^28^. In the present study, we determined that the K_m_ for EGCg was approximately 0.6 mM, which was at the same level as that for oxalate, reported as 0.65 ± 0.08 mM^28^. However, the V_max_ for EGCg was two digits smaller than that of oxalate. Furthermore, although sulfate, chloride, hydroxide, and oxalate are transported optimally under alkaline and neutral pH conditions^28^, it was found that EGCg was transported equally under alkaline, neutral, and acidic pH conditions, as assessed by determining EGCg uptake in *Xenopus* oocytes (data not shown). Taken together, these data suggest that there are differences in the mechanisms through which DTDST transports EGCg and other substrates. We also revealed that DTDST did not transport gallic acid, which is the partial structure of EGCg. The substrate specificity suggested that DTDST did not recognize the gallate moiety itself but the entire structure of EGCg.

Previously, Roth *et al*. reported that EGCg was transported by OATP1A2 and OATP1B3^24^. However, these transporters were found not to be expressed in the small intestine^29,30^. In contrast, DTDST is expressed ubiquitously, including in the intestine^31,32^. After short-term catechin-feeding in mice, the upregulation of DTDST in the ileum may therefore account for the observed increase in the bioavailability of EGCg. We speculate that DTDST is also involved in the intestinal absorption of EGCg in humans because a similar increase in the bioavailability of EGCg after repeated catechin intake in humans has been reported^25^. The present kinetic analysis revealed that DTDST transported EGCg with an approximate K_m_ of 0.6 mM, which is sufficient for the intestinal absorption of EGCg from green tea, which usually contains 0.5–1.5 mM EGCg. Therefore, it is possible that EGCg is absorbed from green tea via intestinal DTDST in humans.

ASBT, one of the EGCg transporter candidate molecules selected in the present study that showed no transport activity for EGCg, has been reported to be inhibited by EGCg^33^. We assume that ASBT was upregulated under EGCg-rich condition in order to fulfill its primary role, which is to reabsorb bile acid adequately. Because EGCg affects the activity of a variety of proteins including digestive enzymes and plasma membrane transporters in the small intestine^33–36^, it is possible that they were also upregulated under EGCg-rich conditions in a compensatory manner to the inhibition by EGCg, as well as ASBT. In the case of DTDST, it might have been inhibited by EGCg in a competitive manner, and the compensatory upregulation might have resulted in enhanced EGCg absorption demonstrated in the present study. Further investigations are needed to clarify the mechanism underlying the induced expression of ileal DTDST by catechin feeding.

There are some limitations to our study. First, other potential EGCg transporters may have been overlooked by each process of the shotgun proteomics analysis and the oocyte expression system. In the shotgun proteomics analysis, the protein selection was based only on proteins that were categorized as plasma membrane transporters by the Ingenuity Pathway Analysis software. As for the oocyte expression system, we could not demonstrate the transport activity using each substrate as a positive control, although we confirmed their expression in plasma membrane fraction. Second, we analyzed the EGCg transport activity of DTDST derived from a murine gene, not a human gene. Third, we did not confirm the EGCg transport activity of DTDST *in vivo*. Further investigations are needed to confirm the contribution of DTDST to determine the bioavailability of EGCg before and after short-term catechin feeding.

In conclusion, we identified DTDST as a novel intestinal EGCg transporter by examining the change in bioavailability of EGCg after short-term catechin feeding in mice. Kinetic analyses indicated that the affinity of DTDST to EGCg was reasonable for the absorption of EGCg from green tea. The identification of this intestinal EGCg transporter should be useful for further examining the regulation of the bioavailability of EGCg, which could be utilized to improve the beneficial effects of green tea. Our results will also be useful for future studies elucidating the absorption mechanisms of other widely consumed polyphenols.

## Supplementary information

Supplemental Tables and Figures

## Data Availability

The datasets generated during and/or analyzed during the current study are available from the corresponding author on reasonable request.
