# Supplementary material for: Identification of the Catechin Uptake Transporter Responsible for Intestinal Absorption of Epigallocatechin Gallate in Mice
Source: Sci Rep. 2019 Jul 29;9:11014. doi: 10.1038/s41598-019-47214-4 (PMC6662683; doi:10.1038/s41598-019-47214-4)
Supplement: Supplementary file 1 — Supplemental Tables and Figures [file 41598_2019_47214_MOESM1_ESM.pdf]

Title: Identification of the Catechin Uptake Transporter Responsible for Intestinal Absorption of Epigallocatechin Gallate in Mice.

Authors: Shunsuke Ishii, Hidefumi Kitazawa, Takuya Mori, Noriko Osaki, Aya Kirino, Shun Nakamura, Akira Shimotoyodome, and Ikumi Tamai

Journal: Scientific Reports.

Supplemental Table 1. Restriction sites used for cloning ORFs of EGCg transporter candidate genes into the pSP64 poly(A) vector or modified vectors and linearization.

Protein name	Gene symbol	Cloning site		Linearization site
		5′-end	3′-end	
ZIP14	*Slc39a14*	Hind III	Xba I	[a] Mlu I
ASBT	*Slc10a2*	Pst I	Sac I	EcoR I
CTL4	*Slc44a4*	Hind III	Xba I	Pvu II
DRA	*Slc26a3*	BamH II	Sac I	EcoR I
DTDST	*Slc26a2*	Pst I	Xba I	[b] Bgl II
LAT2	*Slc7a8*	Hind III	Xba I	[b] Bgl II
NBC1	*Slc4a4*	Sal I	Xba I	EcoR I
MNK	*Atp7a*	Pst I	Xba I	[b] Bgl II
KCC3	*Slc12a6*	Sal I	Xba I	[a] Mlu I
MCT1	*Slc16a1*	Pst I	Xba I	EcoR I

[a]Mlu I and [b]Bgl II sites were inserted into the EcoR I (86) site of the pSP64 poly (A) vector.

Title: Identification of the Catechin Uptake Transporter Responsible for Intestinal Absorption of Epigallocatechin Gallate in Mice.

Authors: Shunsuke Ishii, Hidefumi Kitazawa, Takuya Mori, Noriko Osaki, Aya Kirino, Shun Nakamura, Akira Shimotoyodome, and Ikumi Tamai

Journal: Scientific Reports.

Supplemental Table 2. Fold change of intestinal expression levels of all plasma membrane transporter proteins identified in shotgun proteomics analysis.

Protein name	Gene symbol	UniProtKB ID	[a] Fold Change	
			Jejunum	Ileum
MRP7	Abcc10	MRP7_MOUSE	0.6	15.2
ZIP14	Slc39a14	S39AE_MOUSE	1.1	13.4
ASBT	Slc10a2	NTCP2_MOUSE	0.8	7.1
CTL4	Slc44a4	CTL4_MOUSE	0.5	4.9
MDR1A	Abcb1a	MDR1A_MOUSE	1.8	3.7
y+LAT1	Slc7a7	YLAT1_MOUSE	1.9	3.6
NaDC1	Slc13a2	S13A2_MOUSE	3.3	3.1
DRA	Slc26a3	S26A3_MOUSE	1.4	3.0
DTDST	Slc26a2	S26A2_MOUSE	1.3	3.0
LAT2	Slc7a8	LAT2_MOUSE	1.2	2.8
NBC1	Slc4a4	S4A4_MOUSE	1.5	2.8
OSTb	Slc51b	OSTB_MOUSE	1.7	2.8
SVCT2	Slc23a2	S23A2_MOUSE	2.9	2.6
ARF6	Arf6	ARF6_MOUSE	1.5	2.6
NHERF4	Pdzd3	NHRF4_MOUSE	2.4	2.4
MRP2	Abcc2	MRP2_MOUSE	1.8	2.2
BCRP1	Abcg2	ABCG2_MOUSE	1.1	2.2
ZnT1	Slc30a1	ZNT1_MOUSE	2.4	2.2
NHERF3	Pdzk1	NHRF3_MOUSE	2.2	2.2
MNK	Atp7a	ATP7A_MOUSE	1.5	2.1
LAT4	Slc43a2	LAT4_MOUSE	1.9	2.1
KCC4	Slc12a7	S12A7_MOUSE	1.6	2.1
4F2hc	Slc3a2	4F2_MOUSE	2.1	2.1
KCC3	Slc12a6	S12A6_MOUSE	1.4	2.0
MCT1	Slc16a1	MOT1_MOUSE	1.1	2.0
PEPT1	Slc15a1	S15A1_MOUSE	1.5	2.0
MUSEC1	Stxbp2	STXB2_MOUSE	2.2	2.0
KST1	Slc5a11	SC5AB_MOUSE	2.1	2.0
Sterolin-2	Abcg8	ABCG8_MOUSE	2.2	1.9
TAUT	Slc6a6	SC6A6_MOUSE	0.3	1.9
ATP8B1	Atp8b1	AT8B1_MOUSE	1.6	1.9
NBC3	Slc4a7	S4A7_MOUSE	2.1	1.9
Syntaxin-4	Stx4	STX4_MOUSE	1.4	1.9
TM9SF1	Tm9sf1	TM9S1_MOUSE	1.5	1.8
ATP1B1	Atp1b1	AT1B1_MOUSE	1.4	1.8
Syntaxin-7	Stx7	STX7_MOUSE	1.7	1.8
ATPB3	Atp1b3	AT1B3_MOUSE	1.1	1.7
ZnT5	Slc30a5	ZNT5_MOUSE	2.1	1.7
NKCC1	Slc12a2	S12A2_MOUSE	1.1	1.7
OSTa	Slc51a	OSTA_MOUSE	1.3	1.6
PMCA2	Atp2b2	AT2B2_MOUSE	1.8	1.6
Cadherin-17	Cdh17	CAD17_MOUSE	1.8	1.6
TM9SF2	Tm9sf2	TM9S2_MOUSE	1.5	1.5
Pantophysin	Sypl1	SYPL1_MOUSE	1.8	1.5
GLUT1	Slc2a1	GTR1_MOUSE	1.3	1.5
VAT1	Vat1	VAT1_MOUSE	2.0	1.5
VTI1RP1	Vti1b	VTI1B_MOUSE	4.2	1.5
Basigin	Bsg	BASI_MOUSE	1.5	1.4
TfR1	Tfrc	TFR1_MOUSE	1.3	1.4
ASCT2	Slc1a5	AAAT_MOUSE	0.4	1.3
Hephaestin	Heph	HEPH_MOUSE	1.6	1.3
Syndet	Snap23	SNP23_MOUSE	1.4	1.3
ALDP	Abcd1	ABCD1_MOUSE	1.5	1.2
LDLR	Ldlr	LDLR_MOUSE	1.3	1.2
ZIP7	Slc39a7	S39A7_MOUSE	1.5	1.2
MRP3	Abcc3	MRP3_MOUSE	1.2	1.2
Syntaxin-3	Stx3	STX3_MOUSE	1.2	1.1
MUNC18c	Stxbp3	STXB3_MOUSE	1.5	1.1
NaPi2b	Slc34a2	NPT2B_MOUSE	0.3	1.1
GLUT2	Slc2a2	GTR2_MOUSE	1.5	1.1
SMCT	Slc5a8	SC5A8_MOUSE	16.7	1.1
CTL1	Slc44a1	CTL1_MOUSE	1.3	1.0
V-ATPase subunit A	Atp6v1a	VATA_MOUSE	1.6	1.0
ATP1A1	Atp1a1	AT1A1_MOUSE	1.4	1.0
SEC61G	Sec61g	SC61G_MOUSE	1.6	0.9
AP3b1	Ap3b1	AP3B1_MOUSE	1.3	0.9
ORCTL2	Slc22a18	S22AI_MOUSE	1.2	0.8
SGLT4	Slc5a9	SC5A9_MOUSE	2.2	0.8
Sterolin-1	Abcg5	ABCG5_MOUSE	1.8	0.8
B0AT1	Slc6a19	S6A19_MOUSE	1.1	0.8

CNT2	Slc28a2	S28A2_MOUSE	1.1	0.7
EDB	Vamp8	VAMP8_MOUSE	1.4	0.7
ANK	Ankh	ANKH_MOUSE	2.4	0.6
CAT1	Slc7a1	CTR1_MOUSE	1.2	0.6
PIgR	Pigr	PIGR_MOUSE	0.8	0.5
PCFT/HCP1	Slc46a1	PCFT_MOUSE	1.8	0.5
GLUT5	Slc2a5	GTR5_MOUSE	0.7	0.4
CIP1	Slc12a9	S12A9_MOUSE	2.1	0.4
SGLT1	Slc5a1	SC5A1_MOUSE	1.8	0.3
Syntaxin-17	Stx17	STX17_MOUSE	6.3	0.2

[a] Fold Change of the average expression levels in the catechin group to the control group (n = 3/group).

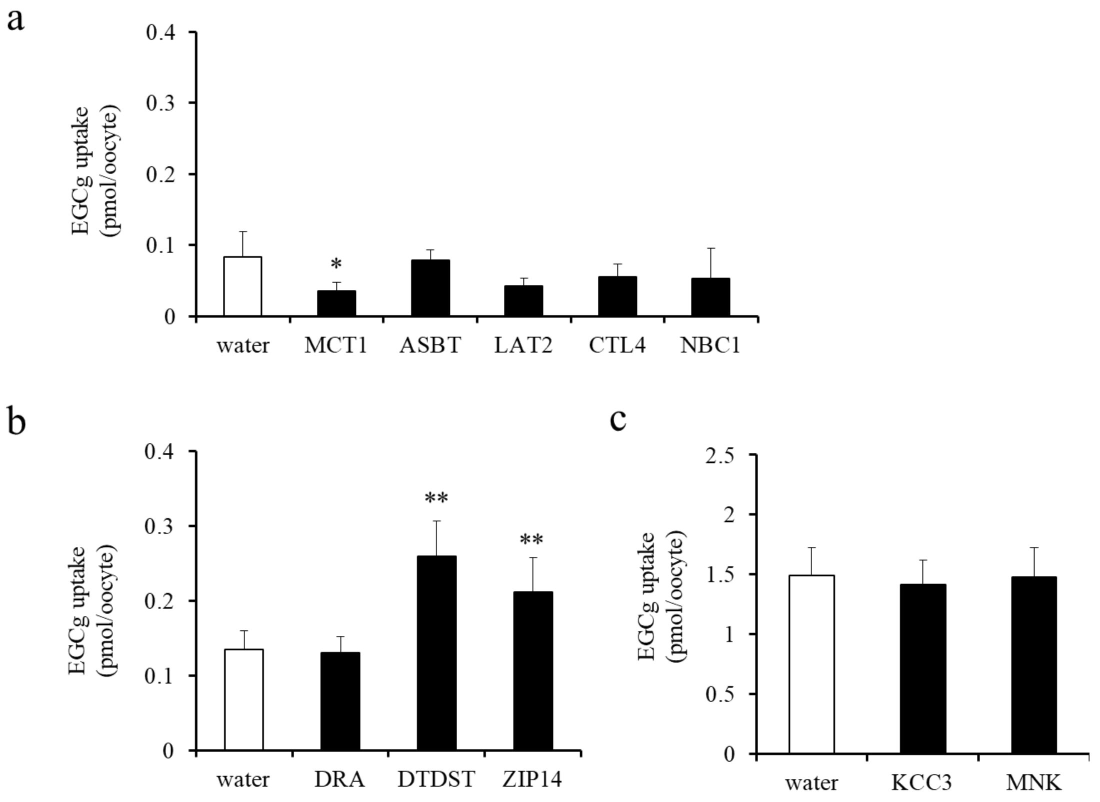

Supplemental Figure 1. (a) Epigallocatechin gallate (EGCg) uptake by *Xenopus laevis* oocytes microinjected with cRNA of monocarboxylate transporter 1 (MCT1), apical sodium-dependent bile acid transporter (ASBT), L-amino acid transporter 2 (LAT2), choline transporter-like protein 4 (CTL4), sodium bicarbonate cotransporter 1 (NBC1), or water (n = 4–5/group) incubated with 100 mM EGCg for 2 hours. Data are presented as mean ± SD. Significant differences as determined by means of Student's t-test are indicated by * ($P < 0.05$) and ** ($P < 0.01$). (b) EGCg uptake by *Xenopus laevis* oocytes microinjected with cRNA of down-regulated in adenoma (DRA), diastrophic dysplasia sulfate transporter (DTDST), zinc transporter 14 (ZIP14), or water (n = 6–8/group) incubated with 100 mM EGCg for 2 hours. Data are presented as mean ± SD. Significant differences as determined by means of Student's t-test are indicated by * ($P < 0.05$) and ** ($P < 0.01$). (c) EGCg uptake by *Xenopus laevis* oocytes microinjected with cRNA of potassium chloride cotransporter 3 (KCC3), Menkes P-type ATPase (MNK), or water (n = 6–8/group) incubated with 500 mM EGCg for 2 hours. Data are presented as mean ± SD. Significant differences as determined by means of Student's t-test are indicated by * ($P < 0.05$) and ** ($P < 0.01$).

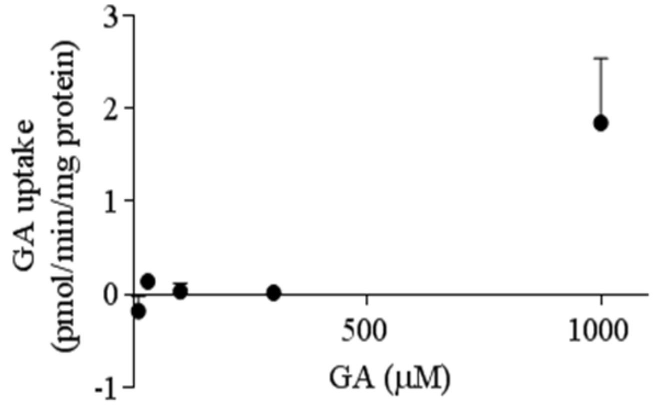

Supplemental Figure 2. Kinetics of gallic acid uptake mediated by DTDST after subtracting the values obtained with mock cells (n = 4). CHO-K1 cells stably expressing DTDST or mock cells were incubated with gallic acid at concentrations ranging from 1 to 1000 μM for 30 min. Data are presented as mean ± SD.
